# Computational Tools for Splicing Defect Prediction in Breast/Ovarian Cancer Genes: How Efficient Are They at Predicting RNA Alterations?

**DOI:** 10.3389/fgene.2018.00366

**Published:** 2018-09-05

**Authors:** Alejandro Moles-Fernández, Laura Duran-Lozano, Gemma Montalban, Sandra Bonache, Irene López-Perolio, Mireia Menéndez, Marta Santamariña, Raquel Behar, Ana Blanco, Estela Carrasco, Adrià López-Fernández, Neda Stjepanovic, Judith Balmaña, Gabriel Capellá, Marta Pineda, Ana Vega, Conxi Lázaro, Miguel de la Hoya, Orland Diez, Sara Gutiérrez-Enríquez

**Affiliations:** ^1^Oncogenetics Group, Vall d’Hebron Institute of Oncology, Barcelona, Spain; ^2^Laboratorio de Oncología Molecular – Centro de Investigación Biomédica en Red de Cancer, Instituto de Investigación Sanitaria San Carlos, Hospital Clínico San Carlos, Madrid, Spain; ^3^Hereditary Cancer Program, Catalan Institute of Oncology, Institut d’Investigació Biomédica de Bellvitge, Hospitalet de Llobregat, Barcelona, Spain; ^4^Program in Molecular Mechanisms and Experimental Therapy in Oncology (Oncobell), Institut d’Investigació Biomédica de Bellvitge, Hospitalet de Llobregat, Barcelona, Spain; ^5^Centro de Investigación Biomédica en Red de Cáncer, Madrid, Spain; ^6^Grupo de Medicina Xenómica-USC, Fundación Pública Galega de Medicina Xenómica-SERGAS, CIBER de Enfermedades Raras, Instituto de Investigación Sanitaria, Santiago de Compostela, Spain; ^7^High Risk and Cancer Prevention Group, Vall d’Hebron Institute of Oncology, Barcelona, Spain; ^8^Medical Oncology Department, University Hospital Vall d’Hebron, Barcelona, Spain; ^9^Area of Clinical and Molecular Genetics, University Hospital Vall d’Hebron, Barcelona, Spain

**Keywords:** hereditary cancer genes, NGS of gene-panel, VUS classification, *in silico* tools, splicing, RNA alteration

## Abstract

*In silico* tools for splicing defect prediction have a key role to assess the impact of variants of uncertain significance. Our aim was to evaluate the performance of a set of commonly used splicing *in silico* tools comparing the predictions against RNA *in vitro* results. This was done for natural splice sites of clinically relevant genes in hereditary breast/ovarian cancer (HBOC) and Lynch syndrome. A study divided into two stages was used to evaluate SSF-like, MaxEntScan, NNSplice, HSF, SPANR, and dbscSNV tools. A discovery dataset of 99 variants with unequivocal results of RNA *in vitro* studies, located in the 10 exonic and 20 intronic nucleotides adjacent to exon–intron boundaries of *BRCA1, BRCA2, MLH1, MSH2, MSH6, PMS2, ATM, BRIP1, CDH1, PALB2, PTEN, RAD51D, STK11*, and *TP53*, was collected from four Spanish cancer genetic laboratories. The best stand-alone predictors or combinations were validated with a set of 346 variants in the same genes with clear splicing outcomes reported in the literature. Sensitivity, specificity, accuracy, negative predictive value (NPV) and Mathews Coefficient Correlation (MCC) scores were used to measure the performance. The discovery stage showed that HSF and SSF-like were the most accurate for variants at the donor and acceptor region, respectively. The further combination analysis revealed that HSF, HSF+SSF-like or HSF+SSF-like+MES achieved a high performance for predicting the disruption of donor sites, and SSF-like or a sequential combination of MES and SSF-like for predicting disruption of acceptor sites. The performance confirmation of these last results with the validation dataset, indicated that the highest sensitivity, accuracy, and NPV (99.44%, 99.44%, and 96.88, respectively) were attained with HSF+SSF-like or HSF+SSF-like+MES for donor sites and SSF-like (92.63%, 92.65%, and 84.44, respectively) for acceptor sites.

We provide recommendations for combining algorithms to conduct *in silico* splicing analysis that achieved a high performance. The high NPV obtained allows to select the variants in which the study by *in vitro* RNA analysis is mandatory against those with a negligible probability of being spliceogenic. Our study also shows that the performance of each specific predictor varies depending on whether the natural splicing sites are donors or acceptors.

## Introduction

The increasing use of massive parallel sequencing of customized multi-gene panels, for germline clinical testing of hereditary breast and ovarian cancer (HBOC) and Lynch syndrome, is leading to higher detection of genetic variants of unknown significance (VUS).

All exonic or intronic VUS can be potentially spliceogenic by disrupting the *cis* DNA sequences that define exons, introns, and regulatory sequences necessary for a correct RNA splicing process. Specifically, the *cis* DNA elements include: (i) exon–intron boundary core consensus nucleotides (GT at +1 and +2 of the 5′donor site and AG at -1 and -2 of the 3′acceptor site); (ii) intronic and exonic nucleotides adjacent to these invariable nucleotides that are also highly conserved and have been found to be critical for splice site selection: CAG/**GU**AAGU in donor sites and NY**AG**/G in acceptor sites; (iii) branch point and polypyrimidine tract sequence motifs, essential for the spliceosome complex formation; (iv) intronic and exonic sequences that act as splicing enhancers (ISE and ESE) or silencers (ISS and ESS), regulatory motifs that are usually bound by serine/arginine (SR)-rich proteins and heterogeneous nuclear ribonucleoproteins (hnRNPs), respectively (Cartegni et al., 2002; Soukarieh et al., 2016; Abramowicz and Gos, 2018). A nucleotide change in any of these elements could lead to incorrect splice site recognition, creating new ones or activating the cryptic ones, resulting in aberrant transcripts and in non-functional proteins associated with disease such as hereditary cancer.

Interestingly, it has recently been described that hereditary cancer genes (including some HBOC and Lynch genes) are enriched for spliceogenic variants (Rhine et al., 2018). This finding highlights the importance of both the identification and the functional interpretation of variants causing RNA alterations in hereditary cancer genes. In HBOC syndrome and Lynch Syndrome, the clinical classification of VUS is essential since carriers of pathogenic variants may benefit from cancer prevention and risk-reducing strategies, make informed decisions about prophylactic surgery, and benefit from targeted treatments (Moreno et al., 2016). Conversely, carriers of non-pathogenic variants can be excluded from intensive follow-ups and avoid unnecessary risk-reducing surgery (Eccles et al., 2015).

To detect splice site alterations, *in vitro* splicing assays with patient’s RNA or minigenes are widely used. However, testing all variants detected in the vicinity of exon–intron boundaries can be time consuming and expensive. In consequence, to select variants to be experimentally evaluated, a large number of prediction programs have been developed. These splicing computational tools are based on different premises. The most commonly used are based on Position Weight Matrix (PWM), in which each nucleotide on the splice site sequence is scored and ranked based on its frequency from its aligned consensus sequence (Shapiro and Senapathy, 1987; Desmet et al., 2009). Neural network programs use sets of sequences from databases to identify splicing sites (Reese et al., 1997). Tools based on Maximum Entropy Distribution models take into account the dependencies between nucleotide positions (Yeo and Burge, 2004). Approaches like SPANR (Xiong et al., 2015) use DNA and RNA sequence information and a machine learning method, to predict splicing alterations, enabling the identification of variants affecting *cis* and *trans* splicing factors. Another type of splicing tool has been developed using ensemble learning methods (adaptive boosting and random forest) taking advantage of individual computational tools (Jian et al., 2014a).

Several studies have analyzed the performance of these tools for genes related to cancer and other diseases and report discordant results without a consensus guideline recommending which programs should be used (Houdayer et al., 2008, 2012; Holla et al., 2009; Vreeswijk et al., 2009; Desmet et al., 2010; Théry et al., 2011; Colombo et al., 2013; Jian et al., 2014a; Tang et al., 2016) (**Table 1**). Here, we present an evaluation of the performance of commonly used splicing *in silico* tools, comparing their output with the experimental evidences obtained by RNA *in vitro* analysis of variants detected in HBOC and Lynch syndrome genes. In the first phase of the study, we assessed the accuracy of the splicing *in silico* tools with a dataset of RNA *in vitro* outcomes collected from four Spanish cancer genetic units. Subsequently, we validated the best algorithms obtained in the discovery phase, with findings obtained after RNA analysis extracted from different curated databases and reported literature.

**Table 1:** Publications evaluating *in silico* splicing site tools.

Reference	Number of variants	Source of the variants and *in vitro* data	Gene(s)	Region analyzed	Experimental design	Prediction tools evaluated	Accuracy of recommended tools	Consensus guideline
Houdayer et al., 2008	39	^∗^Experimental evidence	*RB1*	±60 nucleotides from an AG/GT site	One evaluation stage	NNSplice, PWM, MES, ASSA, ESEfinder, RESCUE-ESE	NA	Not specifically provided
Holla et al., 2009	18	Experimental evidence	*LDLR*	Intronic: 5′ until +5. 3′ until -16	One evaluation stage	MES, NNSplice, NetGene2	NA	Not specifically provided
Vreeswijk et al., 2009	29	Experimental evidence	*BRCA1/BRCA2*	Intronic: 5’ until +60. 3’ until -20	One evaluation stage	NNSplice, NetGene2, PWM, ASSA, MES, HSF	NA	Not specifically provided
Desmet et al., 2010	623	UMD locus-specific databases, HGMD, and datasets from previous studies	Multiple	Not specifically stated	One evaluation stage	GENSCAN, GeneSplicer, HSF, MES, NNSplice, SplicePort, SplicePredictor, SpliceView, SROOGLE	Invariable position: HSF, MES, SpliceView and SROOGLE 100%. Intronic SS +3, +5 and last exonic position: MES 100%. Other SS intronic position: MES and SplicePort 5’ 76/68% and 3’ 77.27/77.27%	Invariable position: HSF, MES, SpliceView and SROOGLE. Intronic SS +3, +5 and last exonic position: MES. Other SS intronic positions: MES and SplicePort
Théry et al., 2011	53	Experimental evidence	*BRCA1/BRCA2*	Not specifically stated	One evaluation stage	PWM, GeneSplicer, NNSplice, MES, HSF	NA	Not specifically provided
Houdayer et al., 2012	272	Experimental evidence	*BRCA1/BRCA2*	Not specifically stated	One evaluation stage	NNSplice, SSF, MES, ESEfinder, RESCUE-ESE, HSF	Accuracy as AUC: MES: 0.956, SSF-like: 0.914	Sequential MES and SSF
Colombo et al., 2013	24	Experimental evidence	*BRCA1/BRCA2*	Not specifically stated	One evaluation stage	PWM, MES, NNSplice, GeneSplicer, HSF, NetGene2, SpliceView, SplicePredictor, ASSA	NA	HSF and ASSA
Jian et al., 2014b	2,959	HGMD, SpliceDisease database and DBASS. Negative variants from 1000 Genomes Phase 1	Multiple	5’: from -3 to +8. 3’: from -12 to +2	Evaluation of individual tools + new model construction + validation stage	SSF-like, MES, NNSplice, GeneSplicer, HSF, NetGene2, GENSCAN, SplicePredictor, ^∗∗^dbscSNV	SSF-like: 91.1% MES: 89.5%/dbscSNV: 93.3%	SSF-like, MES/dbscSNV
Tang et al., 2016	272	HGMD (damaging variants) and negative variants from 1000 Genomes Phase 1	Multiple	Intronic: 5’ from +3 to +7. 3’ from -3 to -9	One evaluation stage	HSF, MES, NNSplice, ASSP	Accuracy as AUC: MES: 0.878 ASSP: 0.881 HSF: 0.834	MES, ASSP, and HSF combination
Leman et al., 2018	395	Experimental evidence	Multiple	5’: from -3 to +8. 3’: from -12 to +2	Training + evaluation stage	HSF, MES, SSF-like, NNSplice, GS, SPiCE (MES and SSF combination)	SPiCE 95.6%	SPiCE (Th_Se_ threshold with MES and SSF combination)

*^∗^Experimental evidence: experimental *in vitro* RNA results collected specifically for the study, derived from either patient blood cells or minigene assay. ^∗∗^dbscSNV: database containing the adaptive boosting and random forests scores. UMD, Universal Mutation Database; HGMD, the Human Gene Mutation Database; DBASS, Aberrant Splice Database; Splice NNSplice, Site Prediction by Neural Network; PWM, Position Weight Matrix; MES, MaxEntScan; ASSA, Automated Splice-Sites Analyses; HSF, Human Splice Finder; SSF, Splice Site Finder; GS, GeneSplicer; SROOGLE, splicing regulation online graphical engine; ASSP, Alternative Splice Site Predictor; NA, information not available in the paper; SS, splicing site; AUC, area under the curve; Th_*Se*_, optimal sensitivity threshold.*

## Materials and Methods

### Variant Selection

#### Discovery Set

We restricted the study to variants located within the last 10 exonic and 20 first intronic nucleotides from the 5′ splice donor site, and the last 20 intronic and the first 10 exonic nucleotides from the 3′ splice acceptor site (-10 to +20 and -20 to +10, respectively). *BRCA1, BRCA2, MLH1, MSH2, MSH6*, and *PMS2* variants were selected from HBOC and Lynch syndrome patients routinely analyzed for diagnostic purposes. We also included *ATM, BRIP1, CDH1, PALB2, PTEN, RAD51D, STK11*, and *TP53* variants obtained in a research series of *BRCA1* and *BRCA2* negative HBOC patients. Genetic variants with unequivocal experimental evidences showing presence or absence of alterations in the mRNA, were collected from four different Spanish centers: Hospital Universitari Vall d’Hebron (HUVH), Barcelona; Hospital Clínico San Carlos (HCSC) Madrid; Fundación Pública Galega de Medicina Xenomica (FPGMX), Santiago de Compostela; Institut Català d’Oncologia (ICO), Hospital Duran i Reynals, Barcelona.

The variants included in the discovery set were analyzed *in vitro* in carriers and controls. RNA was isolated from whole blood leukocytes or short-term lymphocyte cultures, phytohaemagglutinin stimulated, and treated with and without puromycin. The contributing laboratories used diverse isolation protocols and/or cDNA synthesis strategies following ENIGMA recommendations (Colombo et al., 2014; Whiley et al., 2014). Briefly, the splicing products generated by reverse transcription-polymerase chain reaction (RT-PCR) assays were characterized using agarose gel or capillary electrophoresis in a QIAxcel instrument with QIAxcel DNA High Resolution Kit (QIAGEN) or an Agilent 2100 Bioanalyzer (Agilent), and Sanger sequencing. PCR primers were designed to amplify at least one whole exon 5′ and 3′ flanking the exon harboring the variant of interest. Primer sequences are available upon request.

The study was approved by the Institutional Review Board of each participating center. Patients received genetic counseling and written informed consent was obtained for further genetic and research studies.

#### Validation Set

At this stage, the predictors that presented the best performance alone or in combination, were applied to compare their predictions with the *in vitro* RNA results from the dataset obtained through literature and databases. We chose a collection of variants reported in INSIGHT, ClinVar and published works that were (i) located within the regions defined for the discovery set; (ii) identified in the set of cancer risk genes included above; (iii) experimentally confirmed as spliceogenic and non-spliceogenic in blood samples or with minigene assay at least by RT-PCR, agarose gel and Sanger Sequencing analysis; and (iv) not located at exonic splicing enhancer (ESE) regions with specific experimental evidence of causing splicing alteration.

### *In silico* Splice Tools

A total of six splice-site prediction software programs were selected for this study. Two ensemble prediction scores constructed by Jian et al. (2014a) using adaptive boosting and random forests ensemble learning methods, were extracted from dbscSNV database^1^. Splicing-based Analysis of Variants (SPANR), a computational model of splicing derived from the application of “deep learning” computer algorithms (Xiong et al., 2015) was ascertained by its own web site^2^. Splice Site Finder (SSF-like) (based on Shapiro and Senapathy, 1987), MaxEntScan (MES) (Yeo and Burge, 2004), Splice Site Prediction by Neural Network (NNPLICE) (Reese et al., 1997), and Human Splicing Finder (HSF) (Desmet et al., 2009) accessed through Alamut Visual 2.10 (Interactive Biosoftware). The GeneSplicer program is also included in the splicing module of Alamut, but it was excluded from the study since we noticed it had an exceedingly high missing scores (no estimation was obtained for 30% of the variants analyzed; data not shown), which had also been reported by Jian et al. (2014a). SPANR and dbscSNV do not analyze insertions and deletions and dbscSNV gives estimations for variants only located from -3 to +8 at 5′ and -12 to +2 at 3′ (**Supplementary Table 1**).

To interrogate the splicing prediction tools, we calculated the score variation caused by the variant in the donor site or acceptor site. To do that, we compared the score computed in the wild-type sequence (WT) to the score computed in the variant sequence (VAR) as:

$$\begin{array}{c} \%scorevariation\;=\;(VARscore\;-\;WTscore)/WTscore{)}^{*}100 \end{array}$$

We calculated the % score variation for four out of the six tools (SSF-like, HSF, MES, and NNSPLICE), since dbscSNV and SPANR already provide a score change.

To consider a % score change as a positive prediction of a splicing motif disruption caused by the variant, which would lead to aberrant splicing, we adopted thresholds pre-established in the literature (**Supplementary Table 1**). When two programs were combined, a correct prediction of splicing alteration was considered if at least one of them scored above the threshold. When three, four, five, or six programs were combined, all tools but one had to score above the threshold to indicate splicing alteration.

### Performance Assessment

In the discovery and validation phases, the experimental RNA results for each collected variant were annotated as positive splicing alteration when they unequivocally, verified by gel electrophoresis and Sanger sequencing, lead to: exon skipping, use of a new or cryptic splice site or altered alternative transcript profile. In contrast, a negative splicing alteration was annotated when the *in vitro* RNA result was exactly the same as that obtained in control samples.

For both stages, we calculated the overall accuracy (ratio of overall correct predictions to the total number of predictions), specificity (correct identification of non-spliceogenic variants; true negative rate), and sensitivity (correct identification of deleterious variants; true positive rate). The positive predictive values (PPV, proportion of positive predictions that were true positives), negative predictive values (NPV, proportion of negative predictions that were true negatives), false negative rates (FNR, proportion of false negative detection), and false positive rates (FPR, proportion of false positive detection) were also calculated. Matthews correlation coefficient (MCC) was used to provide a balanced comparison between *in silico* tools.

## Results

### Discovery Set

**FIGURE 1:**
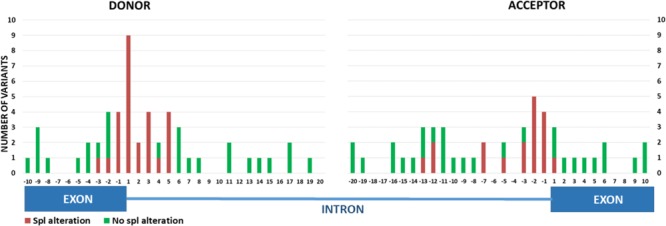
*In vitro* RNA results collected in the **discovery set.** Experimental data are displayed according to variation location. Positive splicing alterations include: exon skipping, use of a new or cryptic splice site or an altered-alternative transcript profile. Negative splicing alteration: *in vitro* RNA result was exactly the same as that obtained in control samples. Spl, splicing.

A total of 99 variants with unequivocal RNA *in vitro* results were studied, located within positions -10 to +20 from the 5′ donor site, and within -20 to +10 from the 3′ acceptor site (**Supplementary Table 2**). Forty-four of the 99 variants generated a splice defect, with 11 and 9 disrupting the canonical GT or AG dinucleotides, respectively. The 24 remaining variants with aberrant splicing were located outside invariable GT or AG positions, with 15 variants altering the 5′ splice site and nine altering the 3′ splice site. Fifty-five variants did not yield an aberrant splicing, all located outside invariant dinucleotides. **Figure 1** displays the number of positive and negative splicing results relative to variant location.

Six *in silico* tools were used to interrogate the 99 variants, and their corresponding % score variation was obtained. These outputs were compared to the experimental RNA results. The respective thresholds pre-established in the literature were adopted for each program (**Supplementary Table 1**).

**Supplementary Table 2** lists the % score variation obtained from each splicing tool used to assess the 99 variants, highlighting which scores were in agreement with the RNA analysis outcome. Of note, seven insertions or deletions were not computed by SPANR and dbscSNV, while estimations for 33 substitutions were not provided by dbscSNV.

**Table 2** shows separately, for 5′ (52 variants), 3′ (47 variants), and both splice sites (global, 99 variants), the results of performance analysis for each one of the tools. The six predictors detected wild type (WT) splice sites in reference sequences for all the genes of interest.

**Table 2:** Performance of the individual *in silico* tools in the discovery dataset.

	Sensitivity	Specificity	Accuracy	MCC	Positive Predictive Value	Negative Predictive Value	False Negative Rate	False Positive Rate	False Discovery Rate	False Omission Rate
**Donor (5′)**										
**HSF**	**100.000**	96.154	**98.077**	**0.962**	96.296	**100.000**	**0.000**	3.846	3.704	**0.000**
**SSF-like**	96.154	96.154	96.154	0.923	96.154	96.154	3.846	3.846	3.846	3.846
**MES**	**100.000**	84.615	92.308	0.856	86.667	**100.000**	**0.000**	15.385	13.333	**0.000**
**dbscSNV**	91.667	90.000	91.176	0.795	95.652	81.818	8.333	10.000	4.348	18.182
**NNS**	92.308	80.769	86.538	0.735	82.759	91.304	7.692	19.231	17.241	8.696
**SPANR**	62.500	**100.000**	81.633	0.677	**100.000**	73.529	37.500	**0.000**	**0.000**	26.471
**Acceptor (3′)**										
**SSF-like**	**100.000**	**89.655**	**93.617**	**0.877**	85.714	**100.000**	**0.000**	**10.345**	14.286	**0.000**
**MES**	**100.000**	86.207	91.489	0.839	81.818	**100.000**	**0.000**	13.793	18.182	**0.000**
**dbscSNV**	93.750	77.778	88.000	0.736	**88.235**	87.500	6.250	22.222	**11.765**	12.500
**HSF**	83.333	82.759	82.979	0.649	75.000	88.889	16.667	17.241	25.000	11.111
**NNS**	88.889	68.966	76.596	0.563	64.000	90.909	11.111	31.034	36.000	9.091
**SPANR**	41.176	88.460	69.760	0.343	70.000	69.697	58.824	11.538	30.000	30.303
**Global (5′ and 3′)**										
**SSF-like**	97.727	92.727	**94.949**	**0.900**	91.489	98.077	2.273	7.273	8.511	1.923
**MES**	**100.000**	85.455	91.919	0.850	84.615	**100.000**	**0.000**	14.545	15.385	**0.000**
**HSF**	93.182	89.091	90.909	0.818	87.234	94.231	6.818	10.909	12.766	5.769
**dbscSNV**	92.500	84.211	89.831	0.767	**92.500**	84.211	7.500	15.789	**7.500**	15.789
**NNS**	90.909	74.545	81.818	0.653	74.074	91.111	9.091	25.455	25.926	8.889
**SPANR**	53.659	**94.118**	76.087	0.533	88.000	71.642	46.341	**5.882**	12.000	28.358

*Results of the performance evaluation is grouped by donor, acceptor or both splice sites. The best performance scores are highlighted in bold. False Discovery Rate represents the rate of false positives of the total of variants positively predicted and False Omission Rate represents the rate of false negatives of the total negative predicted variants. dbscSNV, database consulted for extracting the adaptive boosting and random forests scores.*

On average, predictions for variants located in 5′ regions have higher accuracy (90.98%), sensitivity (90.44%) and specificity (91.28%) compared to those located in 3′ regions (83.74%, 84.52%, and 82.30%, respectively) (**Table 2**). The predictions computed by HSF (with a score change threshold of -2%) were the most accurate and sensitive for variants at donor site, while for variants at acceptor sites or affecting either acceptor or donor sites (global), SSF-like were the most accurate (with a score change threshold of -5%). MES program (with a score change threshold of -15%) showed 100% of sensitivity on all predictions, but its specificity did not reach 87% in any case. In contrast, SPANR program showed the highest values of specificity for predictions of variants at donor site or all variants affecting either at acceptor or donor splice sites, but the lowest values of sensitivity (**Table 2**).

**FIGURE 2:**
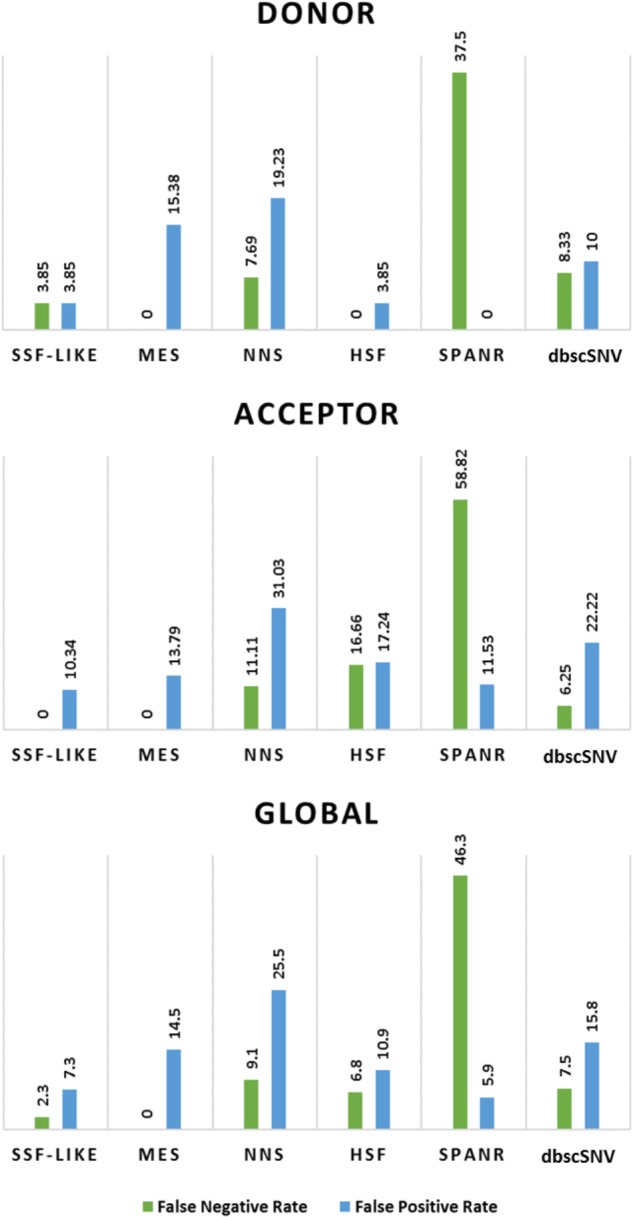
False negative and false positive rates for individual splicing prediction tools in the **discovery set**. dbscSNV, database consulted for extracting the adaptive boosting and random forests scores.

Accordingly, the lowest false negative rates for 5′splice site were reached by the HSF and MES predictors, while at 3′splice sites, the SSF-like and MES predictors obtained the lowest false negative rates (**Table 2** and **Figure 2**). In contrast, SPANR predictor had the highest false negative and the lowest false positive rates in almost all cases (**Table 2** and **Figure 2**). Regarding the estimation of the proportion of negative predictions that were true negatives (NPV), HSF or MES and SSF-like or MES achieved the highest values (100%) for donor and acceptor sites, respectively (**Table 2**).

The accuracy of all possible predictor combinations was further assessed. For 5′ donor splice sites, predictions of HSF alone or HSF together with seven different combinations, SSF-like+SPANR and SSF-like+MES+SPANR reached a 98.08% of accuracy with the highest sensitivity for all the models (100%), obtaining 96.15% of specificity, 0.96 MCC and 100% of NPV (**Supplementary Table 3**). For 3′ splice sites, a sequential combination recommended by Houdayer et al. (2012) using MES as first-line analysis with a cut-off of 15% followed by SSF-like with a 5% threshold achieved the best performance, with a 100% of sensitivity, 96.55% of specificity, 97.87 % of accuracy, 0.96 MCC, and 100% of NPV (**Supplementary Table 4**). However, SSF-like alone and two more combinations including it also showed a 100% of NPV together with 100% sensitivity and high values of accuracy (for predictions at acceptor site, **Supplementary Table 4**). Considering the tool combinations for predicting disruption caused by variants located in any of the two splice sites (global), MES and SSF-like sequential combination achieved the best accuracy with a 96.97% and 0.94 of MCC, followed for two combinations, including SSF-like and MES, which showed 100% sensitivity and 100% of NPV (**Supplementary Table 5**).

### Validation Set

**FIGURE 3:**
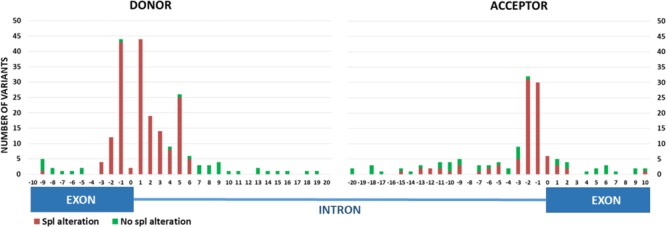
*In vitro* RNA results collected in the **validation set.** Experimental data are displayed according to variation location. Variants located at 0 position are those that affect the invariable dinucleotide positions (GT or AG) plus other contiguous nucleotides. Positive splicing alterations include: exon skipping, use of a new or cryptic splice site or an altered-alternative transcript profile. Negative splicing alteration: *in vitro* RNA result was exactly the same as that obtained in control samples. Spl, splicing.

In order to validate the predictors with the best performance obtained in the discovery set, we analyzed a dataset of 346 variants with RNA *in vitro* results published or detailed in free available databases. At donor region, 210 variants were included, 177 showing *in vitro* splicing alterations (65 at intronic GT positions) and 33 showing no splicing effects (all outside intronic GT) (**Figure 3** and **Supplementary Table 6**). One hundred thirty-six variants were located at the acceptor region, 95 showing splicing alterations (67 of them at intronic AG positions), and 41 with absence of alterations (40 of them outside intronic AG) (**Figure 3** and **Supplementary Table 7**). Only SSF-like and SPANR were able to identify all WT splice sites in reference sequences for all the genes of interest.

We selected for validation, the HSF stand-alone and the combinations HSF+SSF-like and HSF+SSF-like+MES for 5′donor sites (**Supplementary Table 3**), and the SSF-like alone and the sequential MES and SSF combination for 3′acceptor sites (**Supplementary Table 4**), considering sensitivity, accuracy, MCC and NPV scores. We excluded the combinations including SPANR or dbscSNV since they do not provide predictions on insertions and deletions.

**FIGURE 4:**
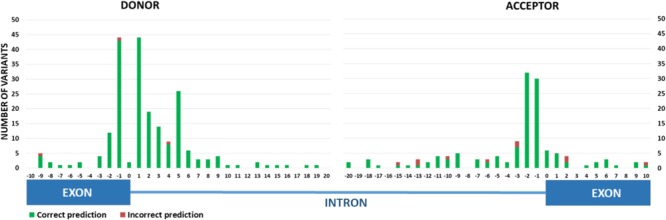
Prediction performance of HSF+SSF-like for donor sites and SSF-like for acceptor sites with variants collected in the **validation set.** Correct prediction: *in silico* and *in vitro* results are concordant. Incorrect prediction: *in silico* and *in vitro* results are discordant.

Overall, the *in silico* predictions in the validation dataset were more accurate for variants with effects on donor splice sites than acceptor sites (**Table 3** and **Figure 4**). These findings were in agreement with those results obtained with the discovery set (**Table 2**).

**Table 3:** Performance with the validation dataset of the best *in silico* tools previously selected from the results at discovery stage.

	Sensitivity	Specificity	Accuracy	MCC	Positive predictive Value	Negative Predictive Value	False Negative Rate	False Positive Rate	False Discovery Rate	False Omission Rate
**Donor**										
**HSF**										
All variants	96.045	90.909	95.238	0.831	98.266	81.081	3.955	9.091	1.734	18.919
Without invariable dinucleotides	94.643	90.909	93.793	0.830	97.248	83.333	5.357	9.091	2.752	16.667
**HSF+SSF-like**										
All variants	**99.435**	**93.939**	**98.571**	**0.946**	**98.876**	**96.875**	**0.565**	**6.061**	**1.124**	**3.125**
Without invariable dinucleotides	**99.107**	**93.939**	**97.931**	**0.941**	**98.230**	**96.875**	**0.893**	**6.061**	**1.770**	**3.125**
**HSF+SSF-like+MES**										
All variants	**99.435**	**93.939**	**98.571**	**0.946**	**98.876**	**96.875**	**0.565**	**6.061**	**1.124**	**3.125**
Without invariable dinucleotides	**99.107**	**93.939**	**97.931**	**0.941**	**98.230**	**96.875**	**0.893**	**6.061**	**1.770**	**3.125**
**Acceptor**										
**MES and SSF-like sequential**										
All variants	91.579	**95.122**	**92.647**	**0.837**	**97.753**	82.979	8.421	**4.878**	**2.247**	17.021
Without invariable dinucleotides	71.429	**95.000**	**85.294**	**0.699**	**90.909**	82.609	28.571	**5.000**	**9.091**	17.391
**SSF-like**										
All variants	**92.632**	92.683	**92.647**	0.832	96.703	**84.444**	**7.368**	7.317	3.297	**15.556**
Without invariable dinucleotides	**75.000**	92.500	**85.294**	0.695	87.500	**84.091**	**25.000**	7.500	12.500	**15.909**

*The best performance scores are highlighted in bold. The atypical *BRCA2* exon 17 native donor site (GC) was not estimated by HSF nor MES, and we have considered it as a failed prediction of the two tools for variants affecting this exon regardless of the *in vitro* splicing effect of the variant. False Discovery Rate represents the rate of false positives of the total of variants positively predicted and False Omission Rate represents the rate of false negatives of the total negative predicted variants.*

The data analysis indicated that for 5′ donor sites the best combinations, with 98.57% accuracy, 99.44% of sensitivity and 96.88% of NPV, are HSF+SSF-like or HSF+SSF-like+MES (**Table 3**) with very slight differences in performance, between the estimations of splicing effects for all variants (including variants placed at invariable dinucleotides) and for the group of variants located outside the two invariable nucleotides. For acceptor sites, the sequential combination of MES and SSF-like (Houdayer et al., 2012) and SSF-like stand-alone reached a performance with the same score of accuracy, 92.65%, but SSF-like showed a highest NPV (**Table 3**). Unlike the donor site, the accuracy of these predictors decreased (to 85.29%) when the variants analyzed did not include those at the two nucleotide invariables (AG) of the 3′ acceptor splice site (**Table 3**). For predictions of variants outside these dinucleotides, the rate of false negatives showed by SSF-like is slightly lower than those rates of MES and SSF-like sequential combination (25% versus 28.57%, respectively, **Table 3**).

## Discussion

The use of massive parallel sequencing in clinical diagnostics is leading to a significant increase in data and the detection of a high number of variants of uncertain significance (VUS) with potential effect on splicing which need interpretation. Therefore, prediction of the effect of DNA sequence variations on splicing using *in silico* tools has become a common approach. Several studies have been published on the performance and reliability of *in silico* predictions of the splicing impact of variants (Jian et al., 2014b). **Table 1** details the results obtained in these studies and shows that the recommendations provided about the most appropriate to be used are not concordant. However, the studies that give clear recommendations, always include one of the HSF, SSF, or MES programs, alternatively.

We have evaluated the reliability of *in silico* splicing effect predictions of six programs (MES, HSF, SSF-like, SPANR, NNSplice, and dbscSNV) comparing their scores with splicing *in vitro* analysis outcomes of variants identified in hereditary cancer related genes. We elaborated the study in two stages, discovery and validation, to identify the best predictors or the best combination for their application in routine clinical testing, taking into account the percentages reached for sensitivity, specificity, accuracy and NPV as well as the score of Mathews Coefficient Correlation (MCC).

In the discovery stage, significant performance differences were appreciated among individual tools (**Table 2**). For global, as well as for 5′, and 3′ splice sites, low accuracies of SPANR and NNSplice contrasted with the high performance achieved by SSF, MES, and HSF, while dbscSNV demonstrated an intermediate accuracy.

At the second stage of our study, we validated the combinations of HSF with SSF-like or HSF+SSF-like+MES as the highest performance for splicing aberrations at donor sites, and SSF-like stand-alone at acceptor sites (**Table 3**). All these results are in agreement with the trend observed in the previous published results, where HSF or SSF or MES outperformed other methods (**Table 1**). Of note, besides high accuracy and sensitivity, these validated tools, combined or as stand-alone, also had high NPV. This is relevant in a clinical setting, since it allows to separate the variants with an extremely low or non-existent probability of being abnormally spliceogenic from those variants in which *in vitro* RNA studies are of interest, with the consequent saving of resources in the laboratory.

All of the three predictors are available through Alamut Visual 2.10 (Interactive Biosoftware, Rouen), allowing a high throughput analysis, which is essential in a massive parallel sequencing annotation pipeline. Yet, in the newest version of Alamut Visual (2.11) the HSF predictor is not included in its splicing module, it is freely available at Human Splice Finder website^3^ or through VarAFT software^4^, which allows the annotation of a large batch of variants. MES program is also freely accessible via web^5,6^, although caution should be taken when obtaining predictions via Alamut or via web, since differences have been reported (Tang et al., 2016). SSF-like tool is currently only accessible through Alamut, yet it has been recently published a free program named Splicing Prediction in Consensus Elements (SPiCE^7^) that combines predictions from SSF-like and MES (Leman et al., 2018). On the other hand, SPANR and dbscSNV are free and could be easily implemented in a pipeline (Xiong et al., 2015; Liu et al., 2017), but these tools are not able to interpret splicing alterations caused by insertion or deletions (6.36% of validation set variants), which represents a limitation for their use compared to the other tools.

Non-canonical GC-AG and AT-AC sequences at the splice site invariant positions occur in 0.56 and 0.09% of the splice site pairs, respectively (Abramowicz and Gos, 2018). In the list of the genes that we analyzed, only six splice sites vary from the canonical splice site GT-AG: *ATM* exon 50 donor site (GC), *BRCA2* exon 17 donor site (GC), *MUTYH* exon 14 donor site (GC), *PALB2* exon 12 donor site (GC), *STK11* exon 2 donor site (AT) and exon 3 acceptor site (AC). In our validation dataset, we only had variants at atypical *BRCA2* exon 17 donor site (GC), and among the studied tools, only SSF-like and SPANR were able to identify these atypical splicing sites and made a prediction for variants located nearby. As the performance of SSF-like is better than SPANR, we suggest the use of SSF-like to analyze these non-canonical splicing sites.

The tools analyzed in this article have only been interrogated to predict alteration at donor and acceptor splice sites. However, alterations in RNA may be produced by variant effects on other factors in *cis* (branch points, polypyrimidine tract, intronic and exonic splicing silencers and enhancers) or create new splice sites or activate cryptic ones. At the stage of validation, the rate of false negative predictions is significantly higher for acceptor sites than for donor sites (**Table 3**). This difference may be due to the greater complexity of the sequence adjacent to the 3′, with the presence of the branch point and the polypyrimidine tract. Therefore, variants located in these two last elements could alter RNA and not be detected as changes in the scores of the splicing sites computed by the predictors. For example, the variant c.1066-6T>G at *ATM* (included in the validation set), which is not predicted correctly by MES and SSF-like sequential combination (**Supplementary Table 7**), alters the polypyrimidine tract causing an aberrant transcript (Dörk et al., 2001).

Likewise, the *BRCA2* exonic variant c.467A>G, located nine nucleotides upstream from the 5′ donor site, causes the loss of these last nine nucleotides, while the HSF and SSF-like predicts that their scores for the native donor splice site of 88.9 and 84.5, respectively, are not changed by the variant, which it is misinterpreted as a false negative (**Supplementary Table 6**). Using some of the tools analyzed in our study to identify enhanced cryptic sites or creation of new splice sites, the variant is predicted to cause a new donor site at nine nucleotides from 5′, in concordance with *in vitro* results: SSF-like indicates a new donor site with a score of 96.9 against 84.5 of the natural splice site, MES 11.1 vs. 9.5 and HSF 98.2 vs. 88.9.

Furthermore, variants located in the exonic regions collected in our study could affect enhancer elements (ESEs) leading to an exon skipping, but they would not be correctly predicted by the analyzed tools. Although variants with specific experimental evidence of suffering this type of alteration were not included in our study, most articles consulted do not explicitly describe or exhaustively exclude the effect of ESEs. As an example, the *BRCA1* c.557C>A altering splicing variant gathered at validation set is not predicted to affect native acceptor site by SSF-like, but specific tools to predict splicing defect caused by regulatory sequence disruption indicates an ESE disturbance: ESRseq score of -1.567 (Ke et al., 2011) and HEXplorer ΔHZ_EI_ = -30.24 (Erkelenz et al., 2014).

Computational tools or programs able to perform predictions on the disruption of all *cis* DNA elements would cover the whole landscape of aberrant RNA splicing yielded by spliceogenic VUS. Theoretically, SPANR is able to detect exon skipping caused by all of the elements above mentioned, although our study indicated that this program has a low performance for at least to predict correctly alterations of donor and acceptor sites (**Table 2**). The HSF predictor accessed via its website^8^, also predicts the impact of genetic variations on branch point elements and has been improved for the identification of natural non-canonical splice sites (Oetting et al., 2018). The breast cancer genes PRIORS probabilities program^9^, gives MES estimations of disruption of natural splice sites and also computes the creation of new donor and acceptor splice sites using NNSplice, yet only for *BRCA1* and *BRCA2* genes (Vallée et al., 2016). However, the accuracy and performance of SPANR, HSF, and PRIORS predictions of variants placed in elements other than natural splice sites has not yet been evaluated.

To our knowledge, our study is the only that evaluates the accuracy of different tools separately for donor and acceptor sites, resulting in different recommendations for each one with high performance (**Table 1**).

One limitation of our study is the use of splicing *in silico* tools through a non-free commercial program, Alamut Visual 2.10, with the uncertainty of whether the predictions obtained through Visual Alamut are the same as those estimated directly by the tools in their respective free access websites. We have confirmed that HSF via web (see footnote 8; data not shown) and MES via SPICE (see footnote 7; **Supplementary Table 8**), at least for native splice sites, provide the same estimations than those provided by Alamut Visual 2.10. However, SSF-like predictions obtained through Alamut Visual 2.10 slightly differ from the predictions ascertained through SPICE (**Supplementary Table 8**). Therefore and considering our findings, we recommend as a free pipeline to use HSF accessed via web and MES via SPICE for donor and acceptor site predictions, respectively.

Another limitation is the higher number of variants causing splicing defects compared to the number of variants causing no splicing alteration in our validation dataset. This bias is due to a tendency to report only variants that cause splicing defects. Some studies, in order to avoid this bias, have included common single nucleotide polymorphisms (SNPs) from control dataset, assuming that they do not cause alterations (**Table 1**). Likewise, reports of RNA *in vitro* effects of variants in the two invariable dinucleotides GT-AG are overrepresented, while those located further from splice junctions are less frequently analyzed.

## Conclusion

In conclusion, to perform *in silico* analysis of VUS potentially affecting natural splice sites in hereditary cancer genes, we recommend the use of the HSF+SSF-like combination (with Δ-2% and Δ-5% as thresholds, respectively) for donor sites and SSF-like (Δ-5%) stand-alone for acceptor sites. These tools have shown in the validation stage a high sensitivity and especially a high NPV. Although the *in vitro* study of RNA remains the gold standard to evaluate the process of splicing, and it is not recommended to use these predictions as the sole source of evidence to make clinical assertions (Richards et al., 2015), our results indicate that these combined tools can be used to filter out VUS with a very low probability of altering splicing without losing true spliceogenic variants that will need deeper experimental validation. Complementing the analysis using specific predictors to identify variants that could affect elements other than splice sites (such as branch points or ESEs), may be useful for the screening of the whole RNA defect landscape. Lastly, it is worth stating that (i) the aim of this work was not to classify variants but to provide an *in silico* algorithm with the highest performance to predict an altered *in vitro* splicing regardless of whether the variants are benign or pathogenic; and (ii) the detection of splicing defect does not automatically denote the pathogenicity of the variant for which a comprehensive qualitative and quantitative RNA analysis is warranted as highlighted in ENIGMA^10^ or ACGM guidelines (Richards et al., 2015) for variant classification.

## Author Contributions

AM-F, LD-L, SG-E, and OD: conception or design of the work. AM-F, LD-L, GM, SB, IL-P, MM, MS, RB, AB, EC, AL-F, NS, and MP: acquisition of data for the work. AM-F, AV, CL, MP, GC, MdH, JB, SG-E, and OD: data analysis and interpretation. AMF, SG-E, and OD: drafting the work. All authors: critical revision of the article and final approval of the version to be published.

## Conflict of Interest Statement

The authors declare that the research was conducted in the absence of any commercial or financial relationships that could be construed as a potential conflict of interest.

## Footnotes

1 https://sites.google.com/site/jpopgen/dbNSFP

2 http://tools.genes.toronto.edu/

3 http://www.umd.be/HSF3/

4 https://varaft.eu/

5 http://genes.mit.edu/burgelab/maxent/Xmaxentscan_scoreseq.html

6 http://genes.mit.edu/burgelab/maxent/Xmaxentscan_scoreseq_acc.html

7 https://sourceforge.net/projects/spicev2-1/

8 http://www.umd.be/HSF3/

9 http://priors.hci.utah.edu/PRIORS/index.php

10 https://enigmaconsortium.org/
